# Remarks on the Use of Quinine in Intermittent and Remittent Fevers

**Published:** 1847-02

**Authors:** L. A. Dugas

**Affiliations:** Professor in the Medical College of Georgia


### RECORD OF MEDICAL SCIENCE.

*Remarks on the use of Quinine in Intermittent and Remittent Fevers.* By L. A. Dugas, M. D., Professor in the Medical College of Georgia.—Having received during the past season a number of communications requesting my views on the use of quinine in the treatment of our autumnal fevers, I beg leave to reply to them through the medium of this Journal.

The fevers of this section of our country being almost exclusively paroxysmal, it may be well to premise, very briefly, my views of their pathology, by which it will be perceived that I regard them as essentially different from those *continued* fevers more commonly encountered in colder latitudes, and which have been denominated Typhoid, Typhus, Follicular enteritis, Dothenenteritis, Jail fever, Ship fever, &c.

Our paroxysmal fevers are either intermittent or remittent at their onset; but, if not arrested, the former may, more or less early, become remittent, or the remittent assume the intermittent type; thus showing them to be only different degrees of the same disease. They both present the same paroxysmal phenomena, that is to say, have regular periods of recurrence or exacerbation, and of declension; they are both preceded and accompanied by a general disturbance, more or less marked, of all the functions of the system, but more especially of those usually termed nervous, as those of sensibility and muscular motion. Lassitude, pains in the limbs, back and head, less of muscular strength, are premonitory and persistent in both. The activity of the circulation, however great, is not continuous as in the phlegmasiæ, but partakes of the same paroxysmal character as the other phenomena. Indeed it may be established as a maxim, that no inflammatory disease even assumes the paroxysmal character, inasmuch as all inflammations pursue an uninterrupted course, whether they terminate in resolution, suppuration or gangrene. Wherever inflammation is exposed to ocular observation, it is never seen suddenly to disappear and to return at stated intervals, or otherwise; but it runs a uniform course which cannot be suddenly modified by the efforts of nature nor by any agent with which we are acquainted. Pure inflammation of internal parts, as pneumonia, pleurisy, acute articular rheumatism, enteritis, &c., observes the same course; there is nothing paroxysmal in these diseases; the febrile action is not attended with daily or periodical exacerbations, but gradually progresses to a certain point, and then gradually declines with the subsidence of the inflammation. Periodicity or the paroxysmal peculiarity is characteristic of the neuroses properly so called, of diseases of the nervous system, which modify the functions of remote organs, and which may be dependent upon congestion, but certainly never upon inflammation. We know of no organ, whose inflammation could furnish us any rational explanation of the varied phenomena of intermittent or of remittent fever. Let us however look to the nervous system for the solution of the problem of these fevers, and all becomes perfectly plain. The languor, lassitude, general and local pains, tremor, modifications of the capillary as well as of the general circulation and of the secretions, and, above all, the abrupt transitions from a normal state to one of great perturbation, and from this again to comparative health, together with the periodical returns of the morbid manifestations—all indicate manifestly great disturbance of that pervading system whose condition is reflected in every part of the body—the nervous system. There is no other system whose impairment could by any possibility yield us the phenomena above related; still less is there any single organ that could by any modification of its condition, produce such general perturbation of the acts of the economy. Intermittents and remittents then are unquestionably the manifestations of deranged innervation; and if arrested sufficiently early will be attended with but little injury to any organ. A continuance of frequent repetition of this derangement, however, may more or less seriously implicate the parenchymatous and secerning structures, inducing inflammatory action, and may even terminate in fatal congestions.

With these views of the pathology of paroxysmal fevers, we are led naturally to the use of such remedies as are calculated to arrest or to modify the perversion of innervation. Theory alone would indicate a resort to any agent known to blunt the nervous sensibilities, and thus to diminish their mobility or tendency to perturbation. Narcotics would present themselves in the first line. Every one knows that opium, morphine, camphor, alcoholic liquors, sulphuric ether, &c., are valuable remedies in intermittent fevers. Indeed a favorite prescription with me in such cases is a combination of 2 parts of sulphuric ether, 1 part of tincture of camphor and 1 part of tincture of opium, of which I give a tea-spoonful in a wine-glass of cold water two hours before the expected paroxysm, and half this quantity again at the expected hour of attack ; the patient remaining in bed during the effect of the remedy. This rarely fails in uncomplicated cases of intermittent fever ; if it does not completely succeed the first day, it will the next. I have frequently averted, or favorably modified even a paroxysm of *remittent* fever by the administration of a full dose of morphine ($\frac{1}{4}$ to $\frac{1}{2}$ gr.) half an hour before the expected exacerbation. But the efficacy of narcotics is not so fully borne out by experience as is that of quinine, an agent which as yet holds a position unique in the materia medica. The most striking peculiarity of quinine is its power to prevent the return of periodical affections, and this appears to me to be effected by blunting the susceptibilities of the nervous system. The senses whose acuteness of perception we can most easily observe, are manifestly blunted. Audition is very soon impaired, and so is vision, if the dose of the remedy be large. The effect of quinine on the heart, in our fevers at least, is unquestionably to diminish the force and frequency of its action, and if the quantity administered be large, a general relaxation, attended with a profuse cold sweat, will be produced, resembling and therefore mistaken by the inexperienced for a collapse of fatal tendency. Having tried it in cases of pure phlegmasia, in pneumonia and acute articular rheumatism, for example, without any aggravation of the febrile action, I cannot regard it as a stimulant.

There is, I believe, no difference of opinion in relation to the value of quinine in the treatment of intermittent fevers. I will, therefore, now confine my remarks to Remittent fevers, comprehending under this term, bilious, malignant, congestive, and country fevers. These are usually preceded by premonitions, which if properly attended to, would enable us to avert their developement with great ease. It is, however, exceedingly rare that medical aid is invoked thus early, and the physician is generally called in only during the first or second strong paroxysm ; often much later. The paroxysm, when once fully developed, will usually run its course despite of any efforts we may use to check it. I therefore generally direct merely a foot-bath, and the free use of cold drinks, as water, lemonade, or soda water, until the period of remission. Should there be, however, such a determination to some vital organ as to threaten serious injury before the equilibrium of the circulation be restored by the subsidence of the exacerbation, I abstract blood with cups to the spine, sometimes (though rarely) deplete from the arm, and urge the use of revulsives, as hot and stimulating pediluvia, and sinapisms to the spine, epigastrium, feet, &c.; if the head be congested, the affusion of cold water to it, continued until the pulse be depressed, and repeated as this reacts, is the most efficacious application I know of. Saline enemata, especially if the bowels are full, should not be omitted, as cathartics will very rarely act during the stage of excitement. If the congestion be attended with cold clammy skin, a small and feeble pulse, and prostration of the vital energies, I advise, in addition to the revulsives, large and repeated doses of the above-mentioned combination of ether, laudanum and camphor, until reaction takes place.

The exacerbation having subsided, our treatment should be directed to the prevention of its return, and my invariable rule is *never to permit the occurrence of another paroxysm after I see the patient.* But, it will be asked, can this rule be carried out? I answer that it can *in the great majority of cases*, and that in those in which we fail to accomplish all we desire, we yet so modify the state of things that success is almost certain on the day following. If we be fully impressed with the belief that the fever being once arrested the patient will rapidly return to health, the importance of the rule cannot fail to be appreciated; and that such is the fact will not for a moment be denied by any who have ever tried the practice we recommend. I repeat, that if all our efforts be directed to the prevention of another paroxysm—if we resolve never to allow a patient to have another exacerbation after we see him, the cure of remittent fevers will almost invariably be effected in a day or two.

In the accomplishment of our resolve, quinine must be regarded as the sheet anchor of our dependence, for although we may resort to other means, these can never be but of secondary value. Nor is it necessary in ordinary cases to use such large quantities of the quinine as are recommended by some. The quantity I use in one remission is usually from 15 to 20 grs., but I have sometimes given 30 or 40 grs.; never more. It is rare that less than 15 grs. will prevent the expected paroxysm. Whatever be the quantity we may estimate as necessary, this should be given in such a manner as to have the system fully under its influence an hour or two before the time of the previous exacerbation, and to continue its influence a couple of hours after this time. If the period of remission be eight hours, we may administer 2 grs. hourly—if it be five hours, we may give 3 grs. hourly—if three hours, 5 grains hourly—and if only one hour, we should give 20 grs. at once, and smaller doses subsequently, if necessary, to insure success. According to my observation the *number* of doses is a matter of but little moment—the *quantity* given in a remission is all important. This will depend upon the violence of the attack, the number of paroxysms that have occurred before we see the patient, and the kind of treatment to which he may have been previously subjected. As a general rule, the quantity should be increased as the period of remission is shortened, and in proportion to the number of paroxysms that have preceded its use. I am inclined to think also that it requires more quinine to prevent a paroxysm in one who has been depleted or acted on by emetics and cathartics than in one who has previously been sujected to no medication. The *convalescence* is certainly more rapid when no debilitating process has been instituted, and health is almost immediately restored if the disease be arrested with quinine on the occurrence of the very first paroxysm. There is some choice in the mode of administration, for the sulphate of quinine will act more slowly if given in powder than in solution, and still more so in pills than in powder. Whenever, therefore, a prompt effect is necessary, the solution should always be preferred. If the stomach will not retain it, it may be thrown up the rectum with a little flax-seed tea or thin starch, in about the same dose as if given by the stomach. In this way it acts remarkably well, and, in the treatment of children, who evince great reluctance to its taste, this mode of administration is peculiarly happy.

But the query is often made: would you give the quinine in cases of remittent fever in which the head is evidently affected,—when there is intense cephalagia, or coma, or delirium? in cases in which the stomach seems implicated—the patient vomiting frequently and rejecting everything he takes? in cases in which the bowels are too loose, or very easily disturbed? in cases in which the liver is either torpid or secretes inordinately? in cases in which one paroxysm runs into the succeeding so completely as scarcely to leave any remission of consequence? I answer, unequivocally, yes—and that the stronger the tendency of the disease to localize itself, the more urgent is the necessity to arrest it; for this tendency will increase with every paroxysm, and cease as soon as their return be checked. Let us always bear in mind that the paroxysms are not *occasioned* by the affection of the head, stomach, bowels, or liver, but, on the contrary, that these are the *consequences* of a deranged innervation and of the paroxsmal condition, and our duty is plain. Let us not be alarmed by the bug-bear inflammation and vitiated secretions, nor be deterred from the use of quinine because some still believe it a stimulant, and our success will very soon eradicate every vestige of former prejudices on this subject. It was not without much difficulty that I succeeded a few years ago in persuading a planter, who had long been in the habit of looking on bilious fever as occasioned by the presence of vitiated or superabundant bile, and who consequently treated his negroes with emetics, cathartics and mercurials, that if he would use quinine at the outset, his hands would be in the field in a few days, instead of losing from ten to fifteen days whenever attacked by fever. And yet, after he had fully satisfied himself of the advantage of the proposed change of treatment, his first observation on meeting me was always--"what becomes of the bile? I am afraid that it is still in the system and will again do mischief?"

In order to illustrate some of the positions I have assumed, I will relate a few cases in which the remission was very light, and the tendency to localization imminent.

On the 12th of October, 1841, I was called to see a lad about 10 years of age, and found him in the height of the second paroxysm of a most violent attack of remittent fever. The pulse was full, strong and active; the heat of the surface intense; he complained of violent head-ache, yet was incessantly tossing himself about the bed in wild delirium; his stomach and bowels were quiet. I had but a few days previous seen a patient about the same age, and in the same neighborhood, succumb (without quinine) in the third paroxysm of a similar attack, and I had every reason to apprehend a similar issue in this case, if another paroxysm were permitted to occur. It was now 2 o'clock, P. M. and the next paroxysm was expected to commence at 8 in the evening. He had taken a cathartic the day before I saw him. I immediately opened a vein, to prevent increased injury to the brain, and abstracted blood pretty freely; then applied a blistering plaster over the dorsal region of the spine, and commenced the use of quinine in doses of 2 grs. every hour. At my evening visit (7 o'clock,) I found him quiet, free from delirium, and with very little fever. Ordered the quinine in doses of 1 gr. hourly through the night. The next morning I found him sitting up without fever, and wishing something to eat. He had no return of fever, took no more medicine, and was perfectly well in a few days. I would remark that the delirium entirely subsided only after he had taken several doses of quinine. I have since given it during delirium, without bleeding, and with equally good effect.

On the 28th October, 1841, I was requested to visit a gentleman, about 45 years of age, on the 5th day of a severe remittent fever. I found him with high fever, lying on his back, and so comatose that it was with considerable difficulty that he could be made to notice questions, to which he would then make incoherent replies. His surface was moist with perspiration, though warm. His pulse was frequent, and somewhat strong, but not sufficiently so to warrant bleeding at so advanced a stage of the case and especially as he was of intemperate habits. He had taken two or three cathartics—and the onset of the next paroxysm was expected in three hours. The case was such, that death must of necessity attend the supervention of another paroxysm. Under these circumstances I ordered 5 grs. quinine in solution every hour, and remained to watch the effects, for I was not at that time as well acquainted with them as at present. Indeed I had not before ventured the use of quinine under a similar determination to the head. The administration of each dose was attended with manifest improvement, so that when the time arrived for the recurrence of the paroxysm, my patient was perfectly lucid, had no stupor, and but little fever. I then left him, with orders to take 1 gr. of the quinine hourly, for twelve hours. On the following morning he was sitting up, without fever, and had none afterwards. A mild laxative was all he took during the rapid convalescence.

During the same month, I attended a girl 8 years of age, whose remittent fever was marked by great gastric irritation, so as to cause her to reject every thing she took; quinine solution administered *per rectum* as readily controlled the disease in this as it did in the above cases.

More recently, I saw a gentleman who had been seized at 9 o'clock A. M. with a chill, which was soon followed by the most intense head-ache, intolerance of light, pain in the back and limbs, as well as at the epigastrum. Being of a sanguineous and plethoric habit, I bled him ; then applied sinapisms to the spine and epigastrium, and prescribed a beverage of cream of tartar and cold water. In the afternoon I found that the fever was still high, that he had vomited repeatedly, was much distressed with nausea, and had been gently purged. The sinapisms were ordered to be repeated, the cream of tartar to be discontinued, and small quantities of iced water to be used to relieve thirst during the night; doses of 5 grs. quinine (in powder) were left, one to be taken in very little water at 4 o'clock the next morning, and repeated every two hours thereafter. I visited him at 8 A. M. and found that the fever had continued high during the night, and remitted only towards morning. He had taken 15 grs. quinine, and now had but little fever, although the nausea still persisted, and had caused him to reject the quinine twice, but which being repeated was finally retained. During this day the febrile exacerbation was much less intense, and he was kept on the use of iced water with a little lime water added to it. On the following morning, the nausea still being troublesome, and, apprehending that the quinine in solution or in powder would be rejected, I gave it to him in pills, 4 grains every two hours until he had taken 16 grains. These were retained, the nausea gradually subsided with the fever, and in the afternoon he was convalescent. He suffered a little from debility, but without further treatment, he was out in a few days. In this case headache and, gastric irritation instead of being increased, *subsided* under the use of quinine.

We are frequently called to cases in which we cannot ascertain the periods of exacerbation and of remission because of the ignorance of the patient or of his attendants, or because those periods are not very strongly defined. In such cases we may safely presume that the remission, if there be any, will occur in the morning, as this is most usually the case in these affections. And, under this presumption, I always prescribe about 20 grs. of quinine to be given in 5 gr. doses at intervals of two hours, commencing at the dawn of the next day, without regard to any incidental circumstances. This last injuction is added, because without it the attendant may upon some trivial change assume the responsibility of omitting the remedy at the only time when it might be given with decided advantage. I have known several cases to terminate fatally by such omission to carry out the prescription ; the excuse being that the patient had too much fever, or head-ache, or nausea, &c. We not unfrequently see cases so late that the life of the patient depends entirely on our ability to prevent another paroxysm. No circumstance then must be allowed to interfere with the use of the only certain preventive with which we are acquainted. If it cannot be given in one form it must be given in another; if the stomach rejects it, throw it upon the rectum. At all hazards, give it. If by this course you happen to give the quinine before the remissions have been fully established, it will not increase the fever, but on the contrary lessen its intensity, and consequently hasten the establishment of the remission. We frequently induce a very decided remission in cases in which it has previously been very slight, by the administration of quinine a short time after the fever has reached its acme of intensity, as may be seen by reference to the cases just related.

Having thus far restricted my remarks to the use of quinine in fevers uncomplicated with true phlegmasia or inflammation, it is proper that I say a few words in relation to cases we occasionally encounter, in which genuine phlagmasiæ are complicated with remittent fever or the paroxysmal peculiarity. I allude now specially to the form of Pneumonia and Pleuro-pneumonia which has prevailed more extensively in Georgia and South Carolina, (and perhaps in other southern states) during the last year or two than formerly, and which has been attended with an extraordinary degree of mortality. From what I have seen of such cases, and learnt from my professional brethren here, and elsewhere, I am satisfied that whilst the most striking element of the disease is an inflammation of the pulmonary organs, this is complicated with remittent fever. Indeed they present regular diurnal or tertian exacerbations and remissions of such decided character as to mislead the friends of the patient, and even his physician, into a degree of security which has often proved fatal. Seized with a violent attack of pneumonia, the patient finds himself at once quite ill, but is soon relieved from anxiety by an apparent amelioration of his condition. This continues until the next day, or perhaps the third, when another exacerbation supervenes and rapidly aggravates the condition of the lungs; but the intensity of the symptoms again abates, and the patient is flattered with the hope of approaching convalescence, until a repetition of the paroxysmal affection places his life in imminent peril, if not beyond the reach of remedial means—and all this notwithstanding a vigorous antiphlogistic course of treatment. This disease has been particularly fatal on our plantations, where the daily or tertian amendments of the patient have induced the owners or overseers not to call in medical aid as early as they would have otherwise done.

In all the cases of pneumonia, complicated as above stated, that have come under my observation, I have not hesitated to combine the use of quinine with that of the lancet, antimonials and opiates, and have uniformly had every reason to be entirely satisfied with the result. They do not require, nor can they bear, the same amount of depletion usually regarded as necessary in common pneumonia and pleurisy, and they very rarely yield to antiphlogistics alone. In furnishing my own testimony to the efficacy of the suggested combination, I might add that of other practitioners of distinction, who, entertaining the same views with myself, have met with similar success. It is scarcely necessary to add that the quinine should be given during the periods of remission, and as liberally as though there were no organ in a state of inflammation.

I have now freely and without reserve, given my views in relation to the use of quinine in our remittent fevers—and in lauding, as I have done, its efficacy, I cannot but apprehend that the charge of ultraism will be preferred against me by those who are still unacquainted with its properties. Be this as it may, I fear nothing from the test of time and experience, and will be amply compensated for the temporary odium, if this article will induce any who may have been backward in the use of quinine to give it a fair trial under the circumstances here recommended. It should be borne in mind, however, that we occasionally meet, even in this latitude, cases of typhoid fever, or of enteritic fever, in which quinine possesses no peculiar efficacy. But these fevers do not present the paroxysmal type, and can therefore be easily distinguished from those in which it is useful.—*Southern Med. and Sur. Jour.*

